# Children who are both wasted and stunted are also underweight and have a high risk of death: a descriptive epidemiology of multiple anthropometric deficits using data from 51 countries

**DOI:** 10.1186/s13690-018-0277-1

**Published:** 2018-07-16

**Authors:** Mark Myatt, Tanya Khara, Simon Schoenbuchner, Silke Pietzsch, Carmel Dolan, Natasha Lelijveld, André Briend

**Affiliations:** 1 Brixton Health, Llawryglyn, Powys, Wales, UK; 2 Emergency Nutrition Network, Oxford, UK; 3 MRC Elsie Widdowson Laboratory, Cambridge, UK; 4 Action Against Hunger USA, New York, USA; 5 Department for Population Health, London School of Hygiene and Tropical Medicine, London, UK; 6 No Wasted Lives, Action Against Hunger UK, London, UK; 7 School of Medicine, Centre for Child Health Research, University of Tampere, Tampere, Finland; 8 Department of Nutrition, Exercise and Sports, University of Copenhagen, Copenhagen, Denmark

**Keywords:** Wasting, Stunting, Multiple anthropometric deficits, Anthropometry, Mortality, Prevalence

## Abstract

**Background:**

Wasting and stunting are common. They are implicated in the deaths of almost two million children each year and account for over 12% of disability-adjusted life years lost in young children. Wasting and stunting tend to be addressed as separate issues despite evidence of common causality and the fact that children may suffer simultaneously from both conditions (*WaSt*). Questions remain regarding the risks associated with *WaSt*, which children are most affected, and how best to reach them.

**Methods:**

A database of cross-sectional survey datasets containing data for almost 1.8 million children was compiled. This was analysed to determine the intersection between sets of wasted, stunted, and underweight children; the association between being wasted and being stunted; the severity of wasting and stunting in *WaSt* children; the prevalence of *WaSt* by age and sex, and to identify weight-for-age z-score and mid-upper arm circumference thresholds for detecting cases of *WaSt*. An additional analysis of the WHO Growth Standards sought the maximum possible weight-for-age z-score for *WaSt* children.

**Results:**

All children who were simultaneously wasted and stunted were also underweight. The maximum possible weight-for-age z-score in these children was below − 2.35. Low WHZ and low HAZ have a joint effect on WAZ which varies with age and sex. *WaSt* and “multiple anthropometric deficits” (i.e. being simultaneously wasted, stunted, and underweight) are identical conditions. The conditions of being wasted and being stunted are positively associated with each other. *WaSt* cases have more severe wasting than wasted only cases. *WaSt* cases have more severe stunting than stunted only cases. *WaSt* is largely a disease of younger children and of males. Cases of *WaSt* can be detected with excellent sensitivity and good specificity using weight-for-age.

**Conclusions:**

The category “multiple anthropometric deficits” can be abandoned in favour of *WaSt*. Therapeutic feeding programs should cover *WaSt* cases given the high mortality risk associated with this condition. Work on treatment effectiveness, duration of treatment, and relapse after cure for *WaSt* cases should be undertaken. Routine reporting of the prevalence of *WaSt* should be encouraged. Further work on the aetiology, prevention, case-finding, and treatment of *WaSt* cases as well as the extent to which current interventions are reaching *WaSt* cases is required.

## Background

An estimated 52 million children are wasted (16 million severely wasted) and 155 million children are stunted [1]. Each year, approximately 800,000 deaths are attributable to wasting, 60% of these attributable to severe wasting (SAM), and over one million deaths are attributable to stunting. These figures are based on prevalence estimates from cross-sectional surveys. Wasting is an acute condition and many wasted children will either recover or die within a few weeks. Estimating the number of wasted children present in a population over a given period of time using unadjusted prevalence estimates is likely to miss many new (i.e. incident) cases and significantly underestimate burden and attributable deaths [2]. A recent estimate of the annual global SAM burden that attempts to account for incident cases suggests that 110 million cases per year might be a more accurate estimate for severe wasting alone [3, 4]. Wasting and stunting are also estimated to be associated with the loss of 64.6 and 54.9 million disability-adjusted life years (DALYs) respectively, accounting for 14.8 and 12.6% of the total global DALYs lost for children under five years of age [5]. Recent global analyses reported that substantial progress has been made in reducing the number of stunted children [1, 6]. There has, however, been less progress in reducing the number of wasted children. It seems unlikely that the World Health Assembly goals of a 40% reduction in the prevalence of stuntedness and reducing and maintaining the prevalence of wasting below 5% will be met by the 2025 target date [6].

A number of recent reviews have noted that addressing wasting and stunting as separate issues, as has historically been the case, may not be justified [7–11]. Wasting and stunting are often present in the same populations and there is evidence suggesting that they share many causal factors [12, 13]. Investigations into whether there is a direct causal relationship between wasting and stunting is ongoing and a number of gaps in the evidence base have been identified [14]. One of these evidence gaps relates to the recognition that children can be both wasted and stunted at the same time [6, 15]. The factors leading to this state of ‘concurrence’ are poorly understood but evidence indicates that considerable excess mortality is experienced by children who are concurrently wasted, stunted, and underweight [16].

National estimates of the prevalence and burden of children concurrently wasted and stunted (a condition referred to as *WaSt* throughout this article) have recently been made for 84 countries [17]. Prevalence ranged between zero and 8% and exceeded 5% in 9 of the 84 countries for which estimates were made.

Questions remain regarding mortality and developmental risks associated with *WaSt*, which children are most affected, how *WaSt* cases may be practictably identified at facility and community level, and implications for programs, health policy and planning, and development policy.

This article reports on an analysis of a large database of cross sectional nutritional anthropometry surveys and aims to answer six basic questions: The degree of overlap between being wasted, being stunted, and being underweight; the maximum possible weight-for-age z-score (WAZ) in children with *WaSt*; the direction and strength of the association between being wasted and being stunted; the severity of wasting and stunting in *WaSt* cases; the prevalence of *WaSt* by age and sex; and how *WaSt* cases may be identified. The analysis of the overlap between being wasted, being stunted, and being underweight was prompted by work demonstrating considerable excess mortality in children who were concurrently wasted, stunted, and underweight who have been described as having “multiple anthropometric deficits” [16].

## Methods

### Data management

A database of nutritional anthropometry survey datasets was compiled. Three existing databases were combined. These were a database compiled by Save the Children in 2007 to investigate the implications of replacing the National Centre for Health Statistics (NCHS) reference with the World Health Organisation (WHO) growth standards [18–20], a database compiled by the Community-based Management of Acute Malnutrition (CMAM) Forum and Action Against Hunger in 2015 to describe and map the prevalence of nutritional oedema, and a database of Standard Expanded Nutrition Survey (SENS) datasets from refugee settings compiled by the United Nations High Commissioner for Refugees (UNHCR) in 2016 for monitoring and evaluation (M&E) purposes [21]. All surveys followed the 30-by-30 nutritional anthropometry survey design, the Standardised Monitoring of Assessment of Relief and Transitions (SMART) survey design, or the SENS survey design [21–23].

Survey datasets were transformed into a standard format with the same variables, variable names, variable types, variable lengths, coding schemes, units of measurement, and file format. The scope of the datasets was limited to a small core set of common variables (i.e. cluster identifier, age, sex, weight, height, mid-upper arm circumference (MUAC), and the presence or absence of bilateral pitting oedema). Records with ages below 6 months and above 59 months, with heights below 45 cm and above 120 cm, and with bilateral pitting oedema were censored. Possible duplicate datasets were detected using a checksum algorithm. Duplication was confirmed using record-by-record validation. Confirmed duplicate datasets were removed from the database. Age data were grouped into year-centred age-groups [22]. Z-scores for weight-for-height (WHZ), height-for-age (HAZ), and weight-for-age (WAZ) were calculated using the WHO growth standards [19, 20]. WHO flagging criteria were applied and flagged records censored [19, 20]. The following standard case-definitions were applied to each record:

- Wasted : WHZ < -2.0
- Stunted : HAZ < -2.0
- Underweight : WAZ < -2.0

Case-definitions for being both wasted and stunted (*WaSt*), being wasted but not stunted (wasted only), and being stunted but not wasted (stunted only) were also applied:

- Wasted and stunted (WaSt) : WHZ < -2.0 and HAZ < -2.0
- Wasted but not stunted (wasted only) : WHZ < -2.0 and HAZ ≥ -2.0
- Stunted but not wasted (stunted only) : WHZ ≥ -2.0 and HAZ < -2.0

Datasets were transformed into a standard format and saved as comma-separated-value (CSV) files using the spreadsheet module of *OpenOffice* version 4.1.3. All other data-management tasks were performed using purpose-written *R* language (version 3.4.2) scripts managed using the *R Analytic-Flow* scientific workflow system (version 3.1.1).

### The degree of overlap between wasting, stunting, and underweight in surveys

The degree of overlap between wasted, stunted and underweight was examined using a Venn diagram [24]. The main purpose of this analysis was to determine the magnitudes of the sets:

$$ {\displaystyle \begin{array}{l} Wasted\kern0.5em \cap \kern0.5em Stunted\\ {} and:\\ {} Wasted\kern0.5em \cap \kern0.5em Stunted\kern0.5em \cap \kern0.5em Underweight\end{array}} $$

where ∩ denotes intersection. A ∩ B is the set that contains all members of A that are also members of B (or, equivalently, all members of B that are also members of A). Intersection is the set theory equivalent of the Boolean AND operation.

### The maximum possible WAZ in children with *WaSt* using the WHO growth standards

The maximum expected WAZ for any child aged between 6 and 59 months with *WaSt* was explored using the WHO growth standards. The height required to give HAZ = − 2.0 at all ages between 6 and 59 months was found using the WHO height-for-age reference data. The weight at each height required to give WHZ = − 2.0 was found using the WHO weight-for-height reference data. These weights were then used to find the age specific WAZ for children aged between 6 and 59 months with both WHZ = − 2.0 and HAZ = − 2.0. Separate analyses were performed for males and females.

### The association between being wasted and being stunted

The association between being wasted and being stunted was explored by calculating country-specific odds ratios. The odds ratio (OR) was used as a measure of the strength and direction of association because it is *symmetrical* (i.e. the OR for being wasted given stuntedness and the OR for being stunted given wastedness are identical). Use of the OR avoids the question of what comes before and what comes after, which is not answerable with cross-sectional data. The country-specific ORs were pooled using a random effects meta-analysis [25, 26]. A random effects model was used because WHZ is known to be associated with body-shape which is known to vary between populations [27, 28].

### The severity of wasting and stunting in *WaSt* cases

The severity of wasting in wasted only and *WaSt* children, and the severity of stunting in stunted only and *WaSt* children were compared. Robust estimators of location and scale were calculated and non-parametric tests used because the distributions of both WHZ and HAZ were right-truncated (i.e. at − 2 z-scores) and severely non-normal. Effect sizes were evaluated using the Common Language Effect Size (CLES) statistic [29]. CLES estimates the probability that a random value drawn from one group will be greater than a random value drawn from a second group. The null (i.e. no difference) value is 0.5. A bootstrap estimator of the CLES was used.

### The prevalence of *WaSt* by age and sex

The prevalence of *WaSt* by sex was investigated by calculating a male to female prevalence ratio (i.e. the prevalence of *WaSt* in males divided by the prevalence of *WaSt* in females). Country-specific prevalence ratios were calculated for countries with a total of 30 or more *WaSt* Cases. These were pooled using an inverse variance weighted average fixed effects meta-analysis [26]. The prevalence of *WaSt* by age and sex was investigated using a pyramid plot of data from all datasets from all countries. Age-specific male to female prevalence ratios were also calculated.

### Using weight-for-age and mid-upper arm circumference to detect cases of *WaSt*

Weight-for-age is widely used in growth monitoring and promotion (GMP) programs and in paediatric clinics [30–33]. MUAC is widely used for community-based case-finding and deciding admission into therapeutic feeding programs [34]. The performance of WAZ and MUAC for detecting cases of *WaSt* was examined by calculating the sensitivity and specificity of WAZ and MUAC below systematically varied thresholds for detecting cases of *WaSt*. Thresholds ranged between the 0.5th percentile and the 99.5th percentile of the variable of interest in steps of 0.1 z-scores for WAZ and 1 mm for MUAC. The sensitivity and specificity estimated at each threshold were plotted as receiver operating characteristic (ROC) curves [35]. Areas under the curve (AUC) were estimated for each ROC curve using the trapezoidal rule [36]. Optimal thresholds were identified using the maximum observed value of Youden’s Index [37, 38]. Youden’s Index is a function of both sensitivity and specificity:

$$ J\kern0.5em =\kern0.5em Sensitivity\kern0.5em +\kern0.5em Specificity\kern0.5em -\kern0.5em 1 $$

and is a commonly used measure of diagnostic effectiveness. The maximum value of Youden’s Index occurs at the threshold that optimizes a test’s differentiating ability when equal weight is given to sensitivity and specificity. It occurs at the point on the ROC curve with the maximum vertical distance from the diagonal (chance) line.

All data analysis was performed using purpose-written *R* language (version 3.4.2) scripts managed using the *R Analytic-Flow* scientific workflow system (version 3.1.1).

## Results

A large database of 2426 survey datasets collected in 51 countries between 1992 and 2015 with data from 1,796,991 children was compiled. Table 1 describes the database used in the reported analyses.

**Table 1:** Description of the survey database used in the analysis

Datasets^a^	Number of surveys^a^		2426 surveys from 51 countries
	Country (number)^b^		Afghanistan (43), Albania (1), Angola (22), Bangladesh (28), Benin (7), Burkina Faso (55), Burundi (25), Cameroon (10), Central African Republic (58), Chad (243), Congo - Kinshasa (266), Cote d’Ivoire (49), Djibouti (14), Eritrea (4), Ethiopia (265), Gambia (8), Guatemala (2), Guinea (12), Guinea Bissau (13), Haiti (49), India (8), Indonesia (3), Jordan (4), Kenya (132), Liberia (55), Madagascar (4), Malawi (16), Mali (14), Mauritania (57), Mozambique (13), Myanmar (22), Nepal (15), Niger (38), Nigeria (107), Pakistan (18), Philippines (12), Rwanda (26), Senegal (7), Sierra Leone (58), Somalia (227), South Sudan (140), Sri Lanka (3), Sudan (144), Tajikistan (5), Tanzania (8), Thailand (2), Togo (18), Uganda (84), Yemen (5), Zambia (6), Zimbabwe (1)
	Year of survey (number)		1992 (3), 1993 (15), 1994 (35), 1995 (39), 1996 (27), 1997 (33), 1998 (21), 1999 (26), 2000 (39), 2001 (41), 2002 (55), 2003 (54), 2004 (76), 2005 (99), 2006 (70), 2007 (83), 2008 (143), 2009 (155) 2010 (201), 2011 (773), 2012 (261), 2013 (250) 2014 (340), 2015 (79), Unknown (7)
	Agency (number)		ACF (802), CONCERN (108), FSNAU (207), GOAL (141), IMC (15), IRC (3), MSF (95), Plan International (2), SC (58), TDH (7), UNHCR (347), UNICEF (622), World Vision (18), Zerca y Lejos (1)
Children^c^	Number of children		1,796,991
	Sex	Males	909,099 (50.6%)
		Females	887,892 (49.4%)
	Age	Minimum	06 months
		1st Quartile	18 months
		Median	30 months
		Mean	31 months
		3rd Quartile	44 months
		Maximum	59 months
	Country (number)^a^		Afghanistan (47,813), Albania (892), Angola (17,191), Bangladesh (14,554), Benin (7841), Burkina Faso (41,467), Burundi (14,604), Cameroon (8530), Central African Republic (36,161), Chad (145,506), Congo (Kinshasa) (226,767), Cote d’Ivoire (23,990), Djibouti (5257), Eritrea (2281), Ethiopia (167,368), Gambia (6721), Guatemala (608), Guinea (9487), Guinea Bissau (7131), Haiti (39,465), India (5145), Indonesia (1735), Jordan (1517), Kenya (86,018), Liberia (32,686), Madagascar (3156), Malawi (15,998), Mali (10,901), Mauritania (36,617), Mozambique (4417), Myanmar (14,322), Nepal (8844), Niger (48,995), Nigeria (65,738), Pakistan (14,098), Philippines (6095), Rwanda (15,559), Senegal (8421), Sierra Leone (62,914), Somalia (234,982), South Sudan (96,225), Sri Lanka (2573), Sudan (114,113), Tajikistan (4297), Tanzania (5290), Thailand (1795), Togo (11,835), Uganda (54,236), Yemen (1781), Zambia (2364), Zimbabwe (690)

^a^Numbers given do not include duplicate datasets

^b^Surveys were from emergency and refugees settings. The specified country of origin may not reflect the nationality or ethnicity of survey respondents

^c^Numbers given are for records remaining after the censoring of records with biologically implausible values using WHO flagging criteria

**Fig. 1:**
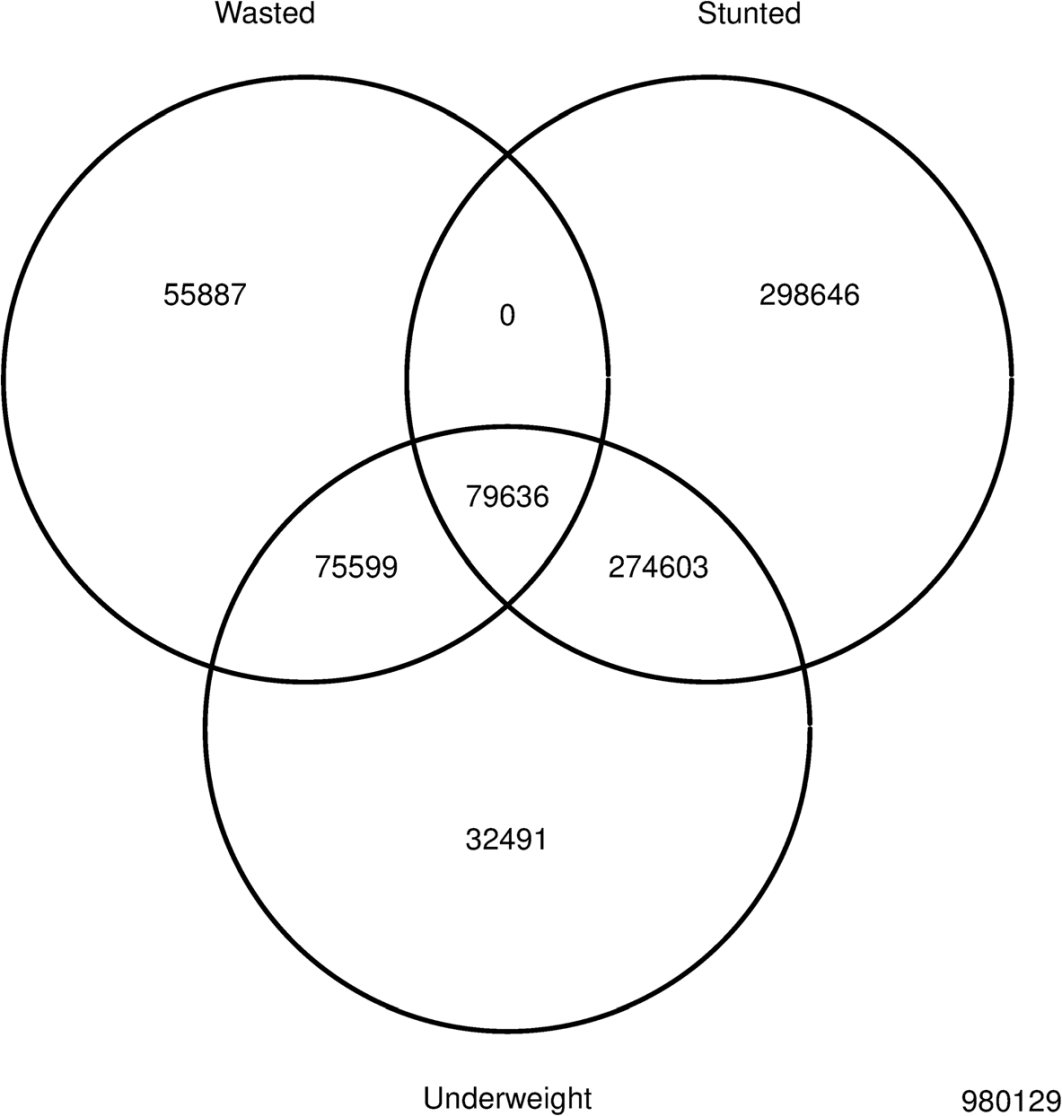
Venn diagram showing the relations between sets of children who are wasted, stunted and underweight in all 51 countries (*n* = 1,796,991)^*^. ^*^ Wasted (weight-for-height z-score < − 2.0), stunted (height-for-age z-score < − 2.0), and underweight (weight-for-age z-score < − 2.0) were defined using z-scores calculated using the WHO growth standards. The number of cases in each division is reported. The zero cell indicates that all *WaSt* cases were also underweight

All children who were simultaneously wasted and stunted were also underweight (see Fig. 1). No child in the database with WHZ < − 2 and HAZ < − 2 had a WAZ ≥ − 2.

**Fig. 2:**
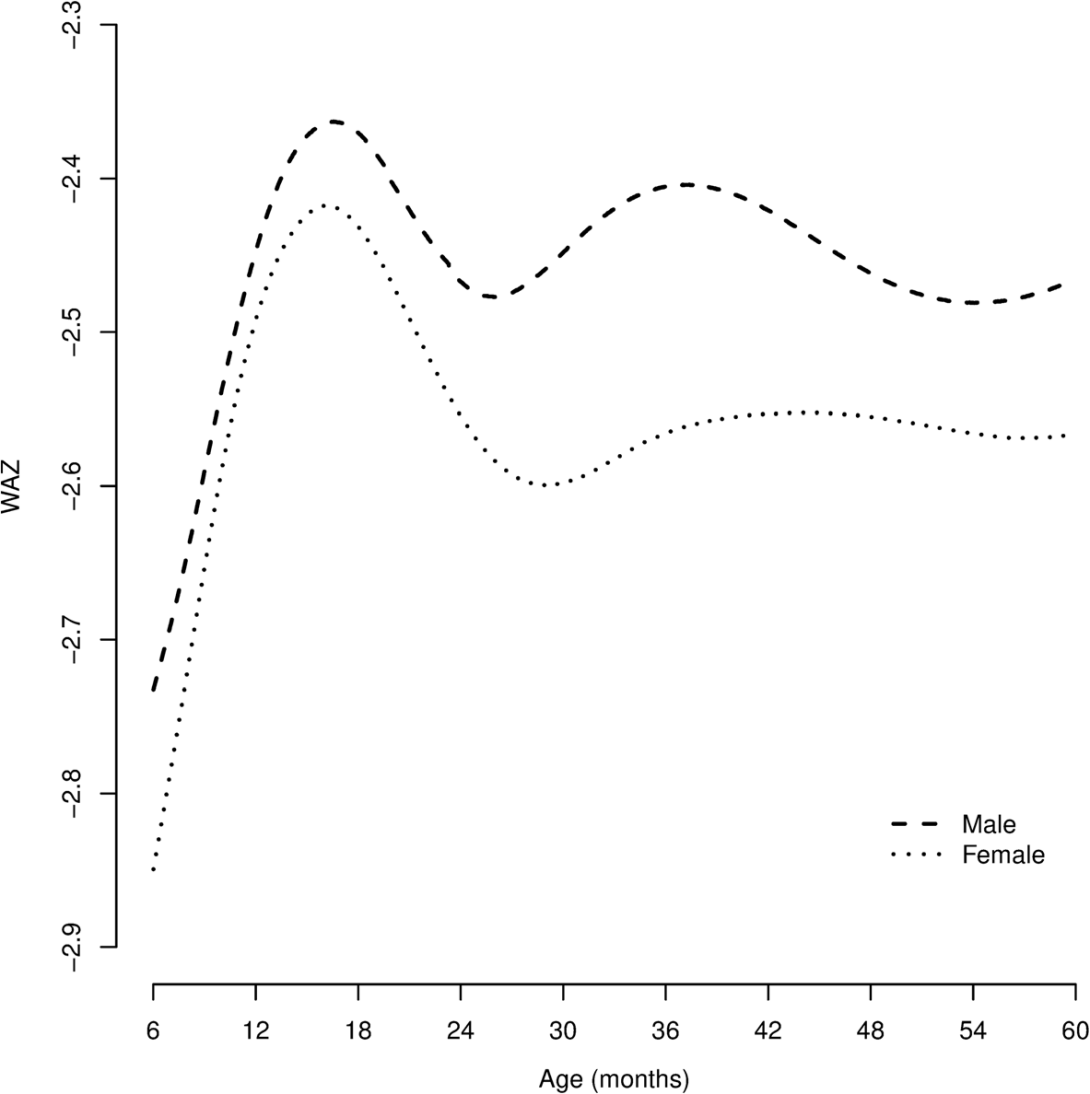
Weight-for-age z-score (WAZ) at different ages for males and females aged between 6 and 59 months with WHZ = − 2.0 and HAZ = − 2.0 calculated using the WHO growth standards. All *WaSt* cases must also be underweight (i.e. present with WAZ < − 2.0)

The maximum WAZ in children who are simultaneously wasted and stunted was WAZ ≈ − 2.36 for males and WAZ ≈ − 2.42 for females when the WHO growth standards are used. Low WHZ and low HAZ have a joint effect on WAZ which varies with age and sex (see Fig. 2).

Significant positive associations between being wasted and being stunted were found in 37 of the 51 countries represented in the database. No significant association was found in 13 countries. A significant negative association was found in one country (Kenya). The pooled estimate of the odds ratio between being stunted and being wasted was 1.40 (95% CI = 1.32–1.49). Detailed results are presented in Table 2.

**Table 2:** Association^a^ between wasted and being stunted in 51 countries

Country	Odds ratio [95% CI]^b^	Country	Odds ratio [95% CI]^b^
Afghanistan	1.22 [1.14, 1.29]	Malawi	1.03 [0.90, 1.17]
Albania	0.78 [0.32, 1.89]	Mali	1.53 [1.36, 1.73]
Angola	1.37 [1.22, 1.53]	Mauritania	1.35 [1.25, 1.45]
Bangladesh	1.37 [1.24, 1.51]	Mozambique	1.31 [1.02, 1.69]
Benin	1.72 [1.45, 2.05]	Myanmar	1.14 [1.03, 1.25]
Burkina Faso	1.74 [1.63, 1.85]	Nepal	1.62 [1.43, 1.85]
Burundi	1.66 [1.45, 1.90]	Niger	1.46 [1.38, 1.54]
Cameroon	1.36 [1.17, 1.59]	Nigeria	1.49 [1.41, 1.57]
Central African Republic	1.59 [1.46, 1.73]	Pakistan	1.00 [0.91, 1.11]
Chad	1.31 [1.27, 1.35]	Philippines	1.53 [1.26, 1.85]
Congo - Kinshasa	0.99 [0.96, 1.02]	Rwanda	1.13 [1.00, 1.27]
Cote d’Ivoire	2.50 [2.24, 2.80]	Senegal	1.76 [1.53, 2.04]
Djibouti	1.31 [1.11, 1.54]	Sierra Leone	1.42 [1.35, 1.50]
Eritrea	0.87 [0.67, 1.13]	Somalia	1.01 [0.98, 1.03]
Ethiopia	1.19 [1.15, 1.22]	South Sudan	1.05 [1.01, 1.09]
Gambia	2.04 [1.73, 2.40]	Sri Lanka	1.84 [1.49, 2.27]
Guatemala	4.97 [0.61,40.62]	Sudan	1.28 [1.24, 1.32]
Guinea	1.71 [1.42, 2.06]	Tajikistan	1.30 [1.07, 1.59]
Guinea Bissau	2.33 [1.90, 2.85]	Tanzania	1.17 [0.91, 1.49]
Haiti	2.27 [2.06, 2.51]	Thailand	1.45 [0.99, 2.12]
India	1.41 [1.22, 1.63]	Togo	2.01 [1.72, 2.35]
Indonesia	1.45 [1.07, 1.97]	Uganda	1.48 [1.38, 1.57]
Jordan	3.42 [1.27, 9.23]	Yemen	1.12 [0.72, 1.72]
Kenya	0.93 [0.89, 0.97]	Zambia	0.80 [0.53, 1.21]
Liberia	1.41 [1.30, 1.53]	Zimbabwe	1.11 [0.50, 2.47]
Madagascar	1.86 [1.45, 2.38]	Pooled OR^c^	1.40 [1.32, 1.49]

^a^The odds ratio (OR) is used here as a measure of the strength and direction of association because it is symmetrical (i.e. the OR for being wasted given stuntedness and the OR for being stunted given wastedness are identical). Use of the OR avoids the question of what comes before and what comes after, which is not answerable with cross-sectional data. OR > 1 is a positive association, OR = 1 is no association, and OR < 1 is a negative association. The distance of the OR from one is a measure of the strength of association

^b^Intervals (ranges) are expressed in ISO 31–11 form. The form [a,b] expresses the interval a ≤ x ≤ b. For example, [1.32,1.49] is used to represent a 95% confidence interval that ranges between 1.32 and 1.49

^c^Estimated using a random effects (DerSimonian-Laird) meta-analysis

The median WHZ in wasted only cases was − 2.47 compared to − 2.52 in *WaSt* cases (*p* < 0.0001) with probability of superiority = 0.522 (95% CI = 0.519–0.525) The median HAZ in stunted only cases was − 2.81 compared to − 2.98 in *WaSt* cases (*p* < 0.0001) with probability of superiority = 0.555 (95% CI = 0.554–0.556).

**Fig. 3:**
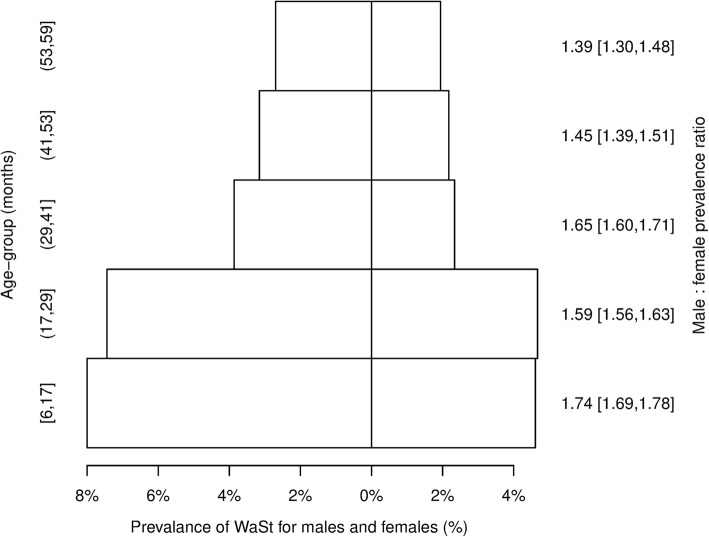
Male and female *WaSt* prevalence by age group in all 51 countries. Intervals (ranges) are expressed in ISO 31–11 form. The form (a, b) expresses the interval a < x ≤ b. For example, (17, 29) is used to represent the set {18, 19, 20, 21, 22, 23, 24, 25, 26, 27, 28, 29} of ages in months. Year-centred age-groups are used. The form (a, b) expresses the interval a ≤ x ≤ b. For example, [1.69, 1.78] is used to represent a 95% confidence interval that ranges between 1.69 and 1.78

The male to female *WaSt* prevalence ratio found in 46 countries for which sufficient data (i.e. *n* ≥ 30 *WaSt* cases) was available are shown in Table 3. The pooled analysis showed the male to female prevalence ratio for *WaST* to be 1.63 (95% CI = 1.63–1.65). Figure 3 shows prevalence of *WaSt* by age and sex found in all 51 countries with age-specific male to female prevalence ratios.

**Table 3:** Male to female *WaSt* prevalence ratio in 46 countries^a^

Country	M:F prevalence ratio [95% CI]^b^	Country	M:F prevalence ratio [95% CI]^b^
Afghanistan	1.39 [1.28,1.50]	Malawi	1.45 [1.23,1.71]
Angola	1.60 [1.39,1.85]	Mali	1.69 [1.40,2.05]
Bangladesh	1.36 [1.20,1.53]	Mauritania	1.70 [1.50,1.93]
Benin	1.44 [1.12,1.85]	Mozambique	2.00 [1.36,2.99]
Burkina Faso	1.85 [1.70,2.03]	Myanmar	1.45 [1.28,1.65]
Burundi	1.49 [1.29,1.71]	Nepal	1.36 [1.17,1.59]
Cameroon	1.93 [1.56,2.40]	Niger	1.49 [1.39,1.60]
Central African Republic	1.57 [1.39,1.76]	Nigeria	1.41 [1.32,1.51]
Chad	1.59 [1.52,1.66]	Pakistan	1.70 [1.48,1.95]
Congo - Kinshasa	1.72 [1.65,1.80]	Philippines	1.54 [1.19,2.01]
Cote d’Ivoire	1.94 [1.66,2.26]	Rwanda	1.44 [1.22,1.69]
Djibouti	1.62 [1.27,2.07]	Senegal	1.87 [1.48,2.37]
Eritrea	1.70 [1.12,2.63]	Sierra Leone	1.53 [1.42,1.66]
Ethiopia	1.87 [1.78,1.97]	Somalia	1.86 [1.78,1.94]
Gambia	2.11 [1.63,2.76]	South Sudan	1.73 [1.62,1.85]
Guinea	1.66 [1.28,2.17]	Sri Lanka	1.02 [0.76,1.37]
Guinea Bissau	1.63 [1.21,2.21]	Sudan	1.71 [1.62,1.79]
Haiti	1.74 [1.51,1.99]	Tajikistan	1.32 [0.99.1.77]
India	1.12 [0.94,1.33]	Tanzania	2.65 [1.80,3.98]
Indonesia	1.35 [0.91,2.02]	Thailand	1.46 [0.84,2.56]
Kenya	1.74 [1.62,1.88]	Togo	1.71 [1.35,2.18]
Liberia	1.58 [1.41,1.77]	Uganda	1.65 [1.50,1.82]
Madagascar	2.47 [1.74,3.54]	Zambia	1.17 [0.59,2.32]
		Pooled PR^c^	1.63 [1.60,1.65]

^a^Countries with fewer than 30 *WaSt* cases (i.e. Albania, Guatemala, Jordan, Yemen, Zimbabwe) were excluded from this analysis

^b^Intervals (ranges) are expressed in ISO 31–11 form. The form [a,b] expresses the interval a ≤ x ≤ b. For example, [1.60,1.65] is used to represent a 95% confidence interval that ranges between 1.60 and 1.65

^c^Calculated using inverse variance-weighted average fixed effects (Cochran) meta-analysis

The sensitivity and specificity for detecting cases of *WaSt* at the thresholds identified using Youden’s Index (i.e. WAZ < − 2.6, MUAC < 133 mm) are shown in Table 4. The areas under the ROC curves are 0.9726 for WAZ and 0.8759 for MUAC.

**Table 4:** Sensitivity and specificity for detecting cases of *WaSt* at thresholds for WAZ and MUAC found using ROC analysis and Youden’s Index

Case-definition^a^	Sensitivity (%)^b^	Specificity (%)^b^
WAZ < − 2.6	98.47 [98.39, 98.56]	91.07 [91.03, 91.11]
MUAC < 133 mm	81.03 [80.76, 81.30]	79.52 [79.46, 79.58]

^a^The case-definitions found using ROC analysis and Youden’s Index

^b^Intervals (ranges) are expressed in ISO 31–11 form. The form [a,b] expresses the interval a ≤ x ≤ b. For example, [98.39, 98.56] is used to represent a 95% confidence interval that ranges between 98.39 and 98.56%

## Discussion

The set:

$$ {\displaystyle \begin{array}{l} Wasted\kern0.5em \cap \kern0.5em Stunted\\ {} and\kern0.5em the\kern0.5em set:\\ {} Wasted\kern0.5em \cap \kern0.5em Stunted\kern0.5em \cap \kern0.5em Underweight\end{array}} $$

are identical to each other. The category of “multiple anthropometric deficits [16]”:

$$ WHZ\kern0.5em <\kern0.5em -2\kern0.5em and\kern0.5em HAZ\kern0.5em <\kern0.5em -2\kern0.5em and\kern0.5em WAZ\kern0.5em <\kern0.5em -2 $$

is not, therefore, different from the *WaSt* category:

$$ WHZ\kern0.5em <\kern0.5em -2\kern0.5em and\kern0.5em HAZ\kern0.5em <\kern0.5em -2 $$

Other workers have noted that “a child cannot simultaneously experience stunting and wasting and not be underweight” but have not formally demonstrate this [39]. It seems sensible to abandon the category of “multiple anthropometric deficits” in favour of *WaSt* which is both simpler and more descriptive. Mortality estimates calculated for “multiple anthropometric deficits” will also apply for *WaSt*. Table 5 shows the pooled hazard ratios for wasted only, stunted only, and *WaSt* from the original work on “multiple anthropometric deficits” [16]. There appears to be a strong interaction effect of wasting and stunting on mortality. This suggests that a common mechanism may link wasting and stunting to an increased risk of death [11]. *WaSt* children are “at a heightened risk of mortality and may benefit most from nutrition and other child survival interventions” [16]. The observed hazard ratio for *WaSt* is comparable to that observed in children with severe wasting defined as WHZ < − 3 (hazard ratio = 12.75, 95% CI = 10.48–15.50) [40]. The high mortality associated with *WaSt* (i.e. pooled hazard ratio = 12.25, 95% CI = 7.67–19.58) compared to not stunted, wasted, or underweight children [16] means that stunted children with moderate wasting should be treated as a priority group for curative interventions.

**Table 5:** Pooled hazard ratios for anthropometric status and all-cause mortality

Anthropometric status	Hazard ratio^a^
Stunted only	1.47 [1.21, 1.78]
Wasted only	2.30 [1.47, 3.60]
Both wasted and stunted (*WaSt*)	12.25 [7.67,19.58]

^a^Pooled hazard ratios and associated 95% confidence limits calculated using data from 10 prospective studies taken from Table 3 of McDonald CM et al. (2013) [16]. The category labelled “*WaSt*” is the same as the category “Wasted, stunted, and underweight” in McDonald CM et al. (2013)

Wasting and stunting were found to be positively and significantly associated with each other in most (i.e. 37 of 51) of the countries from which data were available. The pooled estimate of the odds ratio between stunting and wasting was 1.40 (95% CI = 1.32–1.49). The direction of causality cannot be addressed using cross-sectional data but the results are consistent with *WaSt* being something other than coincidental wasting and stunting.

*WaSt* cases were both more severely wasted than wasted only cases, and more severely stunted than stunted only cases. Effect sizes are small and are probably insufficient to account for the heightened risk of mortality for *WaSt* shown in Table 5. This suggests a multiplicative rather than an additive interaction between wasting and stunting is occurring.

The male to female prevalence ratio for *WaSt* was significantly greater than one in most (i.e. 40 of 46) of the countries from which sufficient data were available. In no country was the male to female prevalence ratio significantly below one and all point estimates were above one. The pooled male to female prevalence ratio was 1.63 (95% CI = 1.60–1.65). *WaSt* appears to be a condition affecting males more than females. The pattern of prevalence by age and sex shown in Fig. 3 was consistently found in country-specific analyses. *WaSt* appears to be a condition that affects children aged below 30 months more than it does older children.

Both WAZ and MUAC performed better than chance at detecting cases of *WaSt*. The performance of WAZ was superior to that of MUAC. Cases of *WaSt* attending, for example, GMP programs and paediatric clinics could be detected with excellent sensitivity and good specificity using WAZ. Cases of *WaSt* could be detected in the community using MUAC but with only moderate sensitivity and specificity.

### Limitations

The narrow scope of the datasets limited the analyses that could be performed. The cross-sectional nature of the data means that no definitive statements about causality could be made. The database was compiled from survey datasets from emergency and post-emergency settings collected for programmatic reasons and provided by agencies that have a policy of archiving survey data and reports, and who are willing to share their data. The database should be treated as ‘found data’ that is subject to a number of selection biases. Results should, therefore, be interpreted with appropriate caution.

## Conclusions

The key findings of the analysis presented in this article are that *WaSt* and “multiple anthropometric deficits” are the same condition, wastedness and stuntedness are positively associated with each other, *WaSt* cases have more severe wasting than wasted only cases. *WaSt* cases have more severe stunting than stunted only cases, *WaSt* is largely a disease of younger children and of males, and that *WaSt* can be detected with excellent sensitivity and good specificity using WAZ.

The use of WAZ < − 2 in the case-definition for “multiple anthropometric deficits” introduces a redundant term into the case-definition and the category should be abandoned in favour of the simpler and more descriptive category of *WaSt*.

There is no pressing need for studies to estimate excess mortality associated with *WaSt* since the available estimates for “multiple anthropometric deficits” may be used [16].

The heightened risk of mortality associated with *WaSt* means that further work on the aetiology, prevention, case-finding, and treatment of children with *WaSt* as well as the extent to which current interventions are reaching children with *WaSt* is urgently required. Work on the mechanisms of the interaction between wasting and stunting on mortality may also prove useful. Work on mortality could be done ethically using data from historical cohort studies.

Treatment of moderate wasting in stunted children should be regarded as a public health priority. Urgent consideration should be given to expanding the admission criteria of therapeutic feeding programs to include children who are *WaSt*. This could be achieved by admitting children with low WAZ identified in paediatric clinics, in GMP programs operating at clinics sites, and in the community. The WAZ threshold used for this purpose could be decided by examination of mortality data from historical cohort studies. The use of MUAC and WAZ is compatible with recent recommendations regarding entry criteria for programs treating SAM in infants aged under six months [41, 42]. How best to identify *WaSt* cases in community settings with no GMP program or a GMP program achieving only low coverage warrants further investigation.

Work on treatment effectiveness, duration of treatment, and relapse after cure for *WaSt* cases should be undertaken. Much of this could be done using existing program data since many *WaSt* cases may already be admitted to CMAM programs [43].

Consideration should be given to encouraging the routine reporting of the prevalence of *WaSt* from nutritional anthropometry surveys, broader surveys (e.g. Multiple Indicator Cluster Surveys and Demographic and Health Surveys), surveillance systems, and other nutrition information systems that collect and report on anthropometric data.

## Abbreviations

ACF: Action Contre la Faim (Action Against Hunger)
AUC: Areas under the curve
CLES: Common Language Effect Size
CMAM: Community-based Management of Acute Malnutrition
CSV: Comma-separated-value
DALY: Disability adjusted life year
ENN: Emergency Nutrition Network
FSNAU: Food Security and Nutrition Analysis Unit
GMP: Growth monitoring and promotion
HAZ: Height-for-age z-score
IMC: International Medical Corps
M&E: Monitoring and evaluation
MSF: Médecins Sans Frontières
MUAC: Mid-upper arm circumference
NCHS: National Centre for Health Statistics
OR: Odds ratio
PPS: Population proportional sampling
ROC: Receiver operating characteristic
SAM: Severe acute malnutrition
SC: Save the Children
SENS: Standard Expanded Nutrition Survey
SMART: Standardised Monitoring of Assessment of Relief and Transitions
TDH: Terre des Hommes
UNHCR: United Nations High Commissioner for Refugees
UNICEF: United Nations Children’s Fund
*WaSt*: Concurrent wasting and stunting
WAZ: Weight-for-age z-score
WHO: World Health Organisation
WHZ: Weight-for-height z-score
